# Xanthine oxidoreductase regulates macrophage IL1β secretion upon NLRP3 inflammasome activation

**DOI:** 10.1038/ncomms7555

**Published:** 2015-03-24

**Authors:** Annette Ives, Johji Nomura, Fabio Martinon, Thierry Roger, Didier LeRoy, Jeffrey N. Miner, Gregoire Simon, Nathalie Busso, Alexander So

**Affiliations:** 1 Rheumatology Laboratory, Centre Hospitalier Universitaire Vaudois, Ave Pierre Decker 4, 1011 Lausanne, Switzerland; 2 Pharmaceutical Development Research Laboratories, Teijin Institute for Bio-Medical Research, Teijin Pharma Limited, Hino, 1918512 Tokyo, Japan; 3 Department of Biochemistry, University of Lausanne, chemin des Boveresses 155, 1066 Epalinges, Switzerland; 4 Infectious Diseases laboratory, Centre Hospitalier Universitaire Vaudois, 1011 Lausanne, Switzerland; 5 Ardea Biosciences, San Diego, 92121 California, USA

## Abstract

Activation of the NLRP3 inflammasome by microbial ligands or tissue damage requires intracellular generation of reactive oxygen species (ROS). We present evidence that macrophage secretion of IL1β upon stimulation with ATP, crystals or LPS is mediated by a rapid increase in the activity of xanthine oxidase (XO), the oxidized form of xanthine dehydrogenase, resulting in the formation of uric acid as well as ROS. We show that XO-derived ROS, but not uric acid, is the trigger for IL1β release and that XO blockade results in impaired IL1β and caspase1 secretion. XO is localized to both cytoplasmic and mitochondrial compartments and acts upstream to the PI3K–AKT signalling pathway that results in mitochondrial ROS generation. This pathway represents a mechanism for regulating NLRP3 inflammasome activation that may have therapeutic implications in inflammatory diseases.

## Abstract (web summary)

Activation of NLRP3 inflammasome requires generation of reactive oxygen species. Here the authors show that microbial or tissue damage-derived signals activate xanthine oxidase, which serves as a critical source of reactive oxygen species for inflammasome activation in macrophages.

## Graphical abstract

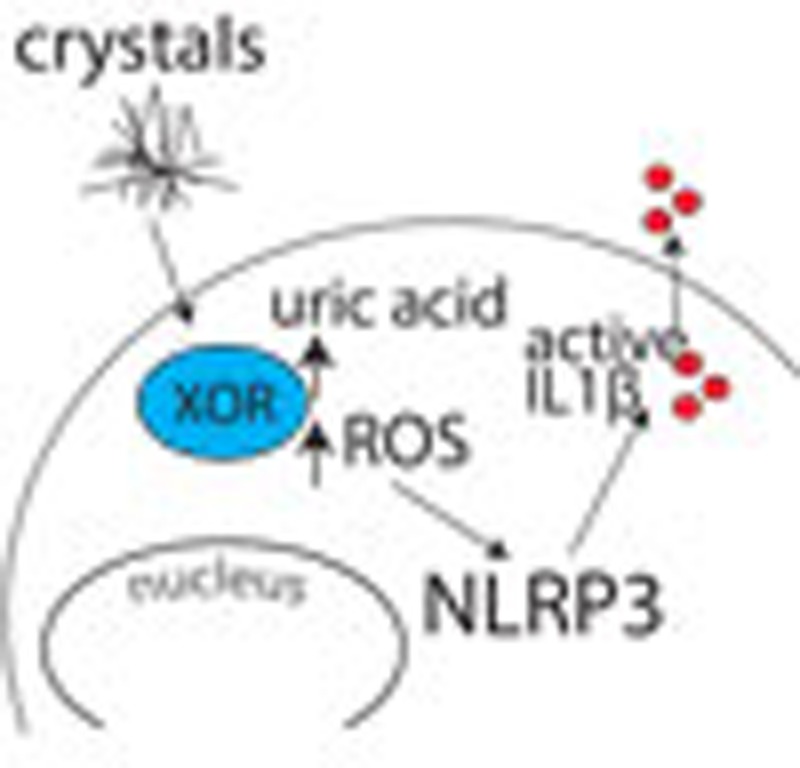

The inflammasome is key in regulating macrophage interleukin (IL)-β secretion in response to pathogen-associated molecular patterns (PAMPs) and damage-associated molecular patterns (DAMPS). The NLRP3 inflammasome, composed of NLRP3, ASC and caspase1, can be activated by soluble and particulate PAMPs (such as lipopolysaccharide (LPS)) and DAMPs (such as urate and calcium crystals, nigericin and adenosine triphosphate (ATP)), resulting in active caspase1 that cleaves proIL1β to the secreted p17kD form of IL1β. Multiple pathways have been found to regulate inflammasome activation: cellular and mitochondrial reactive oxygen species (ROS)^1^, inflammasome translocation to the mitochondria by MAVS^2^, cathepsin B release from phagolysosomes^3^, activity of the cytosolic protein PKR^4^ and changing cytosolic levels of K^+^ (ref. 5) and Ca^2+^ (ref. 6). Among these pathways, ROS generation is shared by a number of different inflammasome triggers, such as LPS, urate crystals and ATP, but the source of ROS has not been clearly established. Initial studies implicated the NADPH oxidase (NOX) complex^7^,^8^, but more recent work found that macrophages derived from knockout mice for the NOX1, NOX2 or NOX4 component of this complex did not impair IL1β secretion, and macrophages derived from patients with chronic granulomatous disease due to mutations of this complex were still capable of secreting IL1β in response to DAMPs^1^,^3^,^9^. Mitochondrial ROS could be an alternative intracellular source, and there is data linking mitochondrial stress to ROS production as well as autophagy^10^,^11^.

Another potential source of cellular ROS is the enzyme xanthine oxidoreductase (XOR), but its role has not been investigated. XOR is a key enzyme in the catabolism of purines into uric acid (UA) that is then further broken down to allantoin in mammals that possess the enzyme uric oxidase (or uricase). The xanthine oxidase (XO) form of XOR utilizes oxygen as a substrate to break down hypoxanthine and xanthine to UA and produces superoxide and hydrogen peroxide as part of the reaction, and is expressed predominantly during cell stress or upon immune activation. A number of clinical and experimental studies have suggested that XOR activity has pro-inflammatory effects and can mediate cardiovascular and endothelial dysfunction^12^,^13^, and inhibition of XOR by allopurinol has been shown to reduce hypertension^14^ as well as improving cardiac function^15^.

UA itself also has both anti-inflammatory as well as pro-inflammatory properties^16^, but the mechanisms linking XOR activity to inflammation remain to be determined. Studies have shown that UA plays a role in innate immune responses, and can act as an adjuvant when released from dying cells, and take part in plasmodium-induced inflammatory responses and the induction of Th_2_ responses in asthma^17^,^18^,^19^,^20^. This raises the question which product, ROS or UA, is responsible for these effects. Allopurinol, an XOR inhibitor, decreased IL1β secretion in response to toll-like receptor (TLR)7/8 stimulation or soluble hemozoin (HZ) administration and inhibited urate production^21^,^22^. However, other studies showed that the addition of uricase failed to block inflammasome-dependent IL1β secretion, suggesting that other mechanisms beside uric-acid production are involved^7^,^23^,^24^.

We hypothesize that XO-dependent generation of ROS mediates NLRP3 inflammasome activation and thus, we studied the role of XOR in DAMP- and PAMP-elicited IL1β responses. Our results demonstrate that XO is a major source of ROS in macrophages and is an important component of innate inflammatory signalling.

## Results

### Crystalline activators require XO to induce IL1β secretion

**Figure 1:**
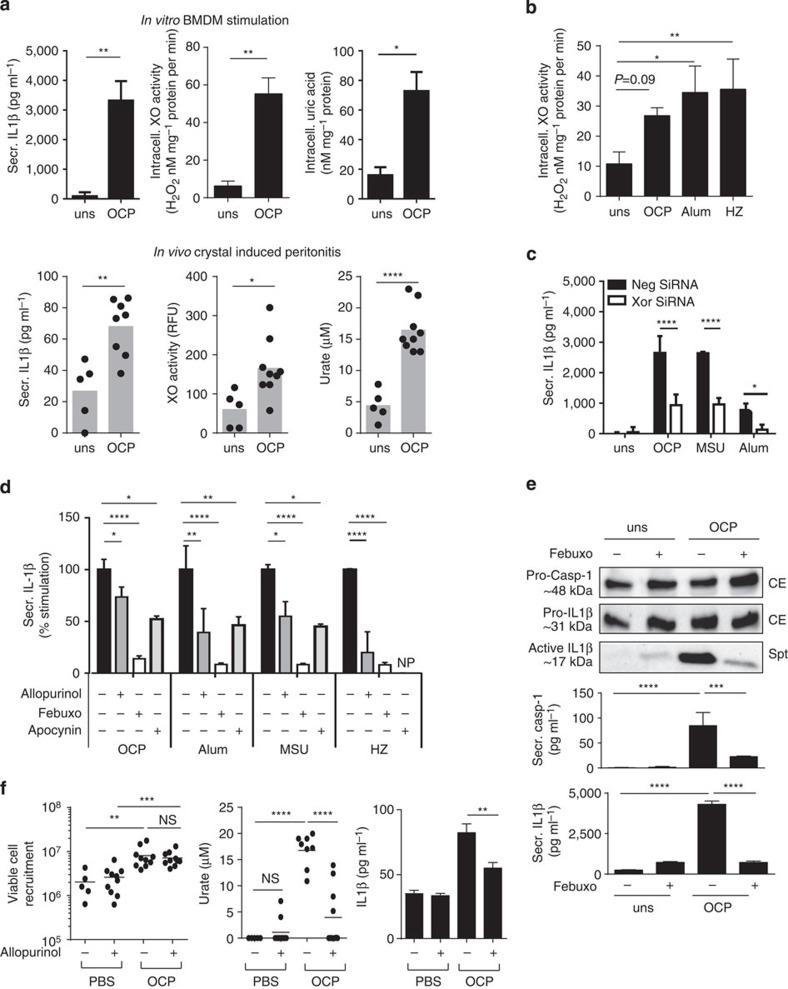
XOR is involved in caspase1-dependent secretion of IL1β in macrophages stimulated with microcrystals. *In vitro* C57BL/6 BMDM (Pam3Cys (P3C) or LPSup primed), and THP1 (PMA primed) were stimulated with OCP and MSU at 250 μg ml^−1^, Alum-500 μg ml^−1^ and HZ-200 μg ml^−1^. IL1β protein was measured by ELISA in supernatants; intracellular XO and UA measured on protein extracts by activity tests. (**a**) Upper panel: P3C primed, OCP-stimulated BMDM. Lower panel: *In vivo* OCP-induced peritonitis. Levels of IL1β, XO and UA measured in peritoneal washes of individual mice. (**b**) XO activity measured in LPSup primed, crystal-stimulated BMDM at 2 h. (**c**) IL1β measured in XOR siRNA, or control NegSiRNA-transfected BMDM that were P3C primed. (**d**) BMDM were pretreated with XOR inhibitors (Allopurinol 250 g ml^−1^, Febuxostat 200 μM) and Apocynin (2.5 mM), extracellular IL1β protein levels quantified and expressed as percentage of stimulation of inhibitor-treated versus non-treated cells. (**e**) Protein sizes of proCaspase1 and proIL1β in cell extracts (CEs), and active IL1β in the supernatants (spts) by western blot, secreted IL1β and caspase1 by ELISA. (**f**) *In vivo*, mice were pretreated with Allopurinol—500 μg per mouse 30 min prior to OCP-1 mg per mouse or PBS control intraperitoneal injection. After 6 h, peritoneal washes were obtained (two pooled experiments). Viable cells were counted and urate, and IL1β measured. Unless stated, experiments were performed at 6 h and data are representative of at least two experiments. Results are expressed as mean±s.e.m. Significance was determined at *P≤0.05, **P≤0.01, ***P≤0.005 and ****P≤0.0001 by analysis of variance (**b**,**d**–**f**) or the *t*-test (**a**,**c**). NP, not performed; NS, not significant.

We previously demonstrated that basic calcium phosphate crystals (including octacalcium phosphate (OCP)) elicit massive IL1β secretion in primed bone-marrow-derived macrophages (BMDMs) via an NLRP3-dependent mechanism *in vitro*^25^. We showed that this process was accompanied by increased intracellular urate levels and XO activity (as assessed by H_2_O_2_ production) (Fig. 1a). *In vivo*, elevated urate and XO activity levels were also correlated to IL1β in peritoneal washes taken from mice during OCP-induced peritonitis (Fig. 1a). Similarly, Alum and HZ stimulation of BMDMs induced XO activity early on during stimulation, at 2 h (Fig. 1b).

To demonstrate that XO activity is required for inflammasome activation and IL1β secretion, we tested the effects of XOR inhibition. Using short interfering RNA (siRNA) to impair XOR expression (knockdown efficiency was determined to be 40±8.4%; *P*≤0.0001, *t*-test by quantitative reverse transcriptase (qRT)–PCR, and 58±3.3%; *P*≤0.005, *t*-test by the XO activity assay) we observed a significant reduction in secreted IL1β in primed and crystal-stimulated BMDM (Fig. 1c). Treatment of primed BMDM with allopurinol or febuxostat (two inhibitors targeting both XO and xanthine dehydrogenase (XDH) forms)^26^,^27^, prior to crystal stimulation, yielded similar results (Fig. 1d). Their inhibitory capacities were similar to that observed with the ROS inhibitor apocynin (Fig. 1d). As febuxostat also inhibited IL1β secretion in human THP1 cells and primary human monocyte-derived macrophages, we confirmed that this was a general mechanism (Fig. 1e; Table 1). Impaired phagocytosis was excluded, as febuxostat did not affect the number of latex beads taken up by Mφ (percentage uptake 63.2±6.6% with febuxostat versus 68.5±1.8% in control cells).

Using the *in vivo* peritonitis model, we confirmed XOR inhibition by allopurinol in OCP-treated mice decreased IL1β and urate levels in the peritoneal lavage, while cellular recruitment remained unchanged (Fig. 1f).

### XOR activity mediates caspase1 processing

As crystal-induced IL1β secretion in macrophages relies on NLRP3 inflammasome assembly and the formation of active caspase1, we examined the role of XOR. We confirmed that febuxostat reduced intracellular caspase1 activity after OCP stimulation (non-treated: 176.63±5.811; febuxostat: 55.20±39.269 relative fluorescence units (RFUs) mg^−1^ of protein; *P*≤0.05, *t*-test). Experiments on primed, but unstimulated BMDMs showed no decrease in the intracellular pool of proIL1β after febuxostat treatment (3,342.50±427.50 and 3,148.75±71.25 pg ml^−1^, respectively) in BMDM at 4 h. Similar results were found in THP1 cells (Fig. 1e). Yet, febuxostat did impact the levels of processed caspase1 and IL1β secreted from the cells (Fig. 1e). Taken together, these results demonstrate that XOR plays an important role in the generation of active caspase1, which allows for IL1β to be cleaved and secreted outside the cell.

We tested the effects of febuxostat on OCP-induced IL1β secretion by human macrophages. Using two different forms of priming (LPS ultrapure and Pam3Cys), OCP crystals induced IL1β release and this was significantly impaired when febuxostat was added (Table 1).

### The effects of XOR are not mediated by urate

**Figure 2:**
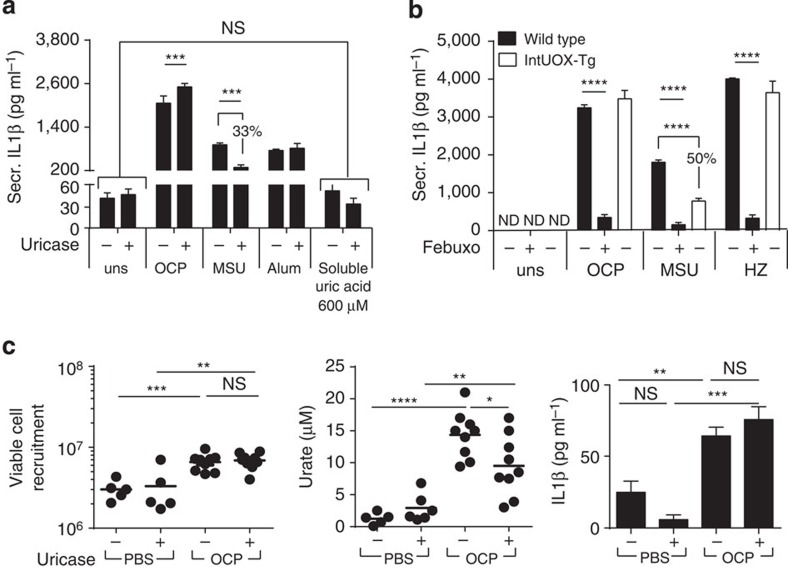
Urate generated by XOR is not the mediator of inflammasome responses in crystal-stimulated macrophages. (**a**,**b**) *In vitro* C57BL/6 or intUOX-Tg-derived BMDM (Pam3Cys primed), pretreated with uricase—10 μg ml^−1^ and XOR inhibitor febuxostat—200 μM, were stimulated with OCP or MSU at 250 μg ml^−1^, Alum-500 μg ml^−1^, HZ-200 μg ml^−1^ or soluble urate at 600  μM. Experiments were performed at 6 h; data are representative of at least two experiments. (**c**) Mice were pretreated with uricase—20 μg per mouse. OCP or PBS injected. After 6 h, peritoneal washes were obtained, viable cells counted, and urate and IL1β measured in cell-free washes (two pooled experiments). Results are expressed as mean±s.e.m. Significance was determined at *P≤0.05, **P≤0.01, ***P≤0.005 and ****P≤0.0001 by analysis of variance. ND, not detectable; NS, not significant.

As UA can act as an immune adjuvant^17^,^28^, we evaluated whether our responses could be due to XOR generation of urate. Despite increased intracellular urate, we did not detect any urate in the culture supernatants, nor did extracellular uricase treatment affect secreted IL1β levels, with the exception of monosodium urate (MSU) stimulation. This was due to crystal degradation (Fig. 2a).

As the accumulated urate was retained within the cell, we stimulated BMDM from transgenic uricase mice (intUOX-Tg) with crystals^28^; no change in IL1β secretion was found (Fig. 2b). This was despite the results that demonstrated that BMDM derived from UOXTg mice did indeed overexpress the *UOX* gene by qRT–PCR as compared with wild-type cells (41.3±6.01 versus 1±0.15, respectively, mean±s.e.m.). Uricase treatment in mice, unlike experiments with allopurinol, failed to impact IL1β levels produced in crystal-induced peritonitis (Figs 1f and 2c).

Finally, as very little is known about the effects of soluble urate and urate transport on immune responses in macrophages, we investigated this using BMDMs. Administration of high concentrations of soluble urate to mimic conditions seen in hyperuricemia in man (at 600 μM), filtered to exclude particulates, did not activate primed BMDM (Fig. 2a). The capacity of BMDM to transport urate across the membrane was also studied. We found BMDM had a limited capacity to take up extracellular soluble urate in a C^14^ -radiolabelled assay (Supplementary Fig. 1a). A positive control hURAT1-transfected cell line confirmed that our assay was indeed functional (Supplementary Fig. 1a). Interestingly, BMDMs express a specific urate transporter gene profile, with only Glut9 being expressed at a significant level. No Urat1 and only weak expression of Abcg2 was detected by qRT–PCR (Supplementary Fig. 1b). Given these results, we did not investigate the protein expression of urate transporters in BMDM. All together, these results exclude a direct effect of soluble urate as the trigger for IL1β release, and favours a mechanism involving XOR-dependent ROS production.

### XO mediates inflammatory signalling by ROS generation

**Figure 3:**
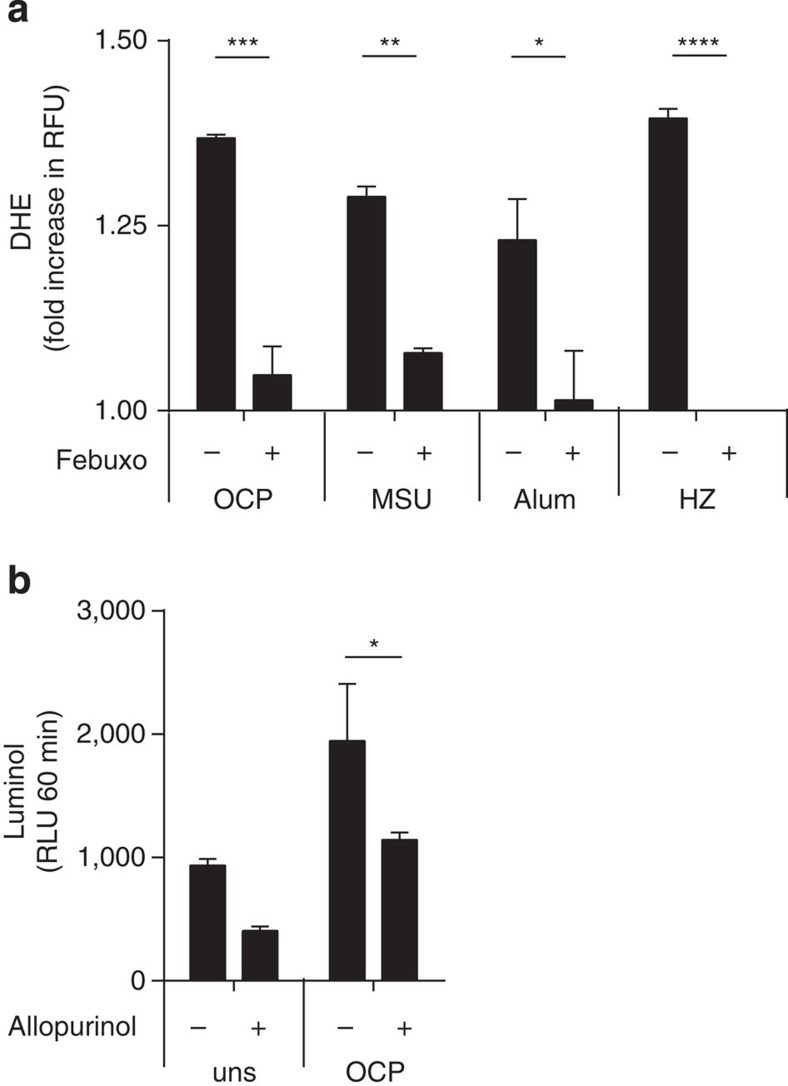
Crystal-induced IL1β secretion is mediated by ROS. *In vitro* BMDM (Pam3Cys primed) were pretreated with XOR inhibitors: febuxostat, 200 μM and allopurinol, 500 μg ml^−1^ and stimulated with OCP or MSU at 250 μg ml^−1^, Alum-500 μg ml^−1^ and HZ-200 μg ml^−1^. (**a**) Superoxide was measured with dihydroethidium (DHE), results expressed as fold increase in relative fluorescence units (RFUs) at *T*=120 min over *T*=0. (**b**) ROS measured by luminol, data obtained by relative luminescence units (RLUs) at *T*=60 min. Data are representative of at least two experiments. Results are expressed as mean±s.e.m. Significance was determined at *P≤0.05, **P≤0.01, ***P≤0.005 and ****P≤0.0001 by the *t*-test for all panels.

Previous studies and our own work demonstrated a role for ROS scavengers in crystal-induced IL1β response, as NAC or apocynin addition impaired IL1β release. Thus, we theorized that XO could be the potential source of ROS in our model. Two different assays were used. Significant reductions of superoxide were observed after febuxostat treatment using the dihydroethidium (DHE) assay, similar to the reductions observed with apocynin (Fig. 3a; Supplementary Fig. 2a), while allopurinol reduced H_2_O_2_ generation by the luminol assay (Fig. 3b).

**Figure 4:**
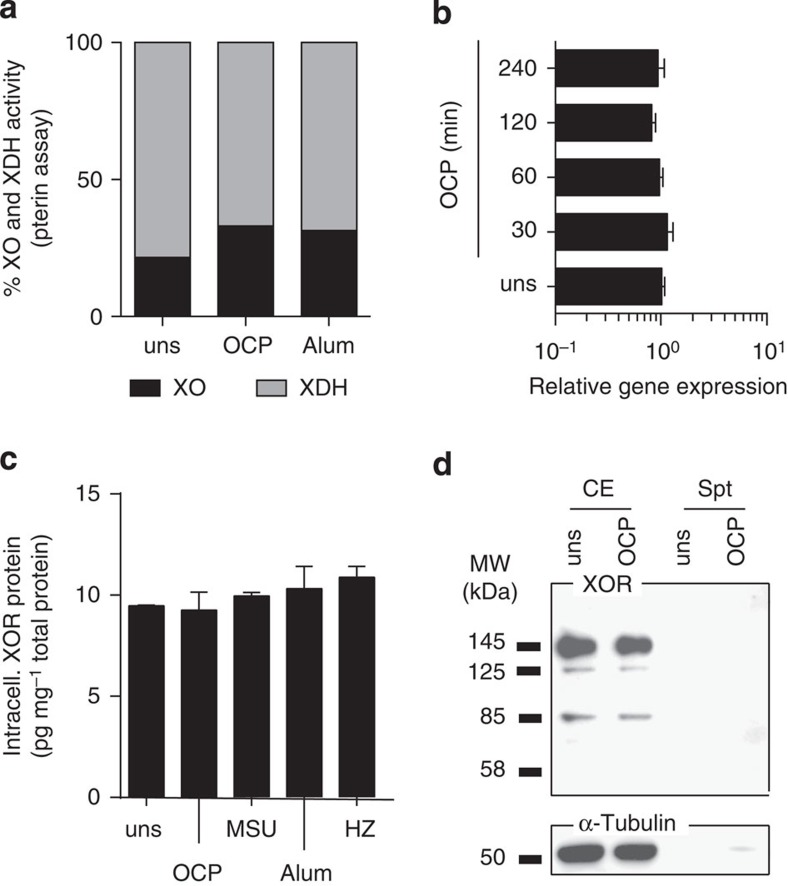
XOR was not modulated at RNA and protein levels. *In vitro* C57BL/6 BMDM (Pam3Cys (P3C) or LPSup primed), and THP1 (PMA primed) were stimulated with OCP and MSU at 250 μg ml^−1^, 500 μg ml^−1^ Alum and 200 μg ml^−1^ HZ. (**a**) The % XO and XDH activity was measured in BMDM cell lysates using the pterin assay; (**b**) RNA transcript level of XOR by qRT–PCR in P3C-primed BMDM. Expression levels were quantified using the 2^ΔΔCT^ method, with *Tbp* as a reference gene. (**c**) Total XOR protein levels in P3C-primed BMDM measured by ELISA. (**d**) THP1 cells stimulated with OCP crystals and the CE, and supernatants blotted against anti-human XOR antibody or α-tubulin.

The relative contribution of XO and XDH activities within the cell was assessed using the pterin assay after crystal stimulation. We found that crystal stimulation increased the percentage of XO. XO activity was measured in the presence of pterin alone, while total XOR activity was measured using both pterin and methylene blue (that replaces the NAD+ electron receptor^29^ (Fig. 4a). Cellular responses to XO-generated superoxide require post-translational modification of the protein, but this can be coupled with increases in the intracellular protein pool. However, no modulation in XOR at the RNA transcript or protein level was observed (Fig. 4b,c). There was also no evidence of proteolytic transformation from the native 145 kDa to the truncated 85-kDa form by western blot (Fig. 4d). This suggests increased XO activity is due to reversible modification of XOR to generate the XO form of the enzyme. In support of this, we found that addition of the antioxidant dithiothreitol to BMDM lysates resulted in a decreased percentage of XO activity relative to total XOR activity in BMDM as compared with non-treated controls (73.7 versus 17.9%, respectively). Altogether, these results suggest that XO activity in crystal-stimulated BMDM was most likely due to reversible sulfhydryl modification of XDH to XO that does not change the molecular weight of the protein.

### XO induces mitochondrial ROS via the PI3K–AKT–mTOR pathway

**Figure 5:**
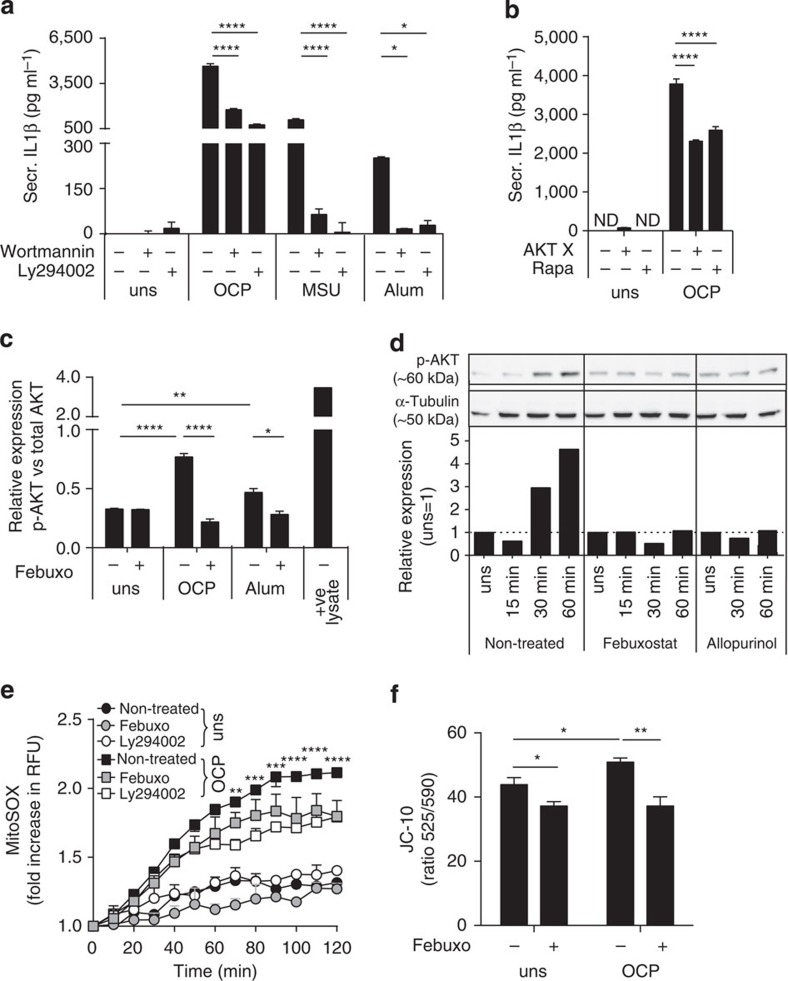
Crystal-mediated XOR activation induces the PI3K–AKT–mTOR pathway and mitochondrial ROS production. *In vitro* BMDM (Pam3Cys primed), and THP1 cells (PMA primed) were pretreated with 200 μM febuxostat, 10 μM Ly294002, 10 μM Wortmannin, 10 μM AKTx, or Rapamycin (Rapa), and stimulated with OCP or MSU at 250 μg ml^−1^, or 500 μg ml^−1^ Alum. (**a**,**b**) IL1β protein secretion measured in BMDM. (**c**) Relative expression levels determined by ELISA of phosphorylated (p-) AKT (ser473) over total AKT levels in BMDM at 1 h. (**d**) THP1 cell extracts analysed for phosphorylated AKT (p-AKT) at ser473, and α-tubulin by western blot. Relative protein levels determined by band intensity quantification, and given as p-AKT over α-tubulin, normalized with unstimulated cells being 1. (**e**) Mitochondrial ROS detected by MitoSOX, results given as RFU at *T*=60 min over *T*=0. (**f**) Mitochondrial potential of BMDM was measured using the ratio of monomeric (Em_525_) to multimeric JC-10 (Em_590_). Experiments were performed at 6 h; data are representative of at least two experiments. Results are expressed as mean±s.e.m. Significance was determined at *P≤0.05, **P≤0.01, ***P≤0.005 and ****P≤0.0001 by analysis of variance for all panels

The PI3K–AKT–mTOR pathway is implicated in ROS-dependent responses to ATP, HZ and basic calcium phosphate crystals^30^,^31^,^32^. Activation of this pathway initiates a signalling cascade that changes cellular metabolism, apoptosis, autophagy and inflammatory responses. We confirmed that crystals induced phosphorylation of AKT in BMDM and THP1, and XOR inhibition decreased the observed levels (Fig. 5c,d). The role of the PI3K–AKT–mTOR pathway was further confirmed, as Ly294002, wortmannin, AKTx inhibitor and rapamycin all significantly decreased IL1β secretion in OCP-stimulated BMDM (Fig. 5a,b). In addition, XO and the PI3K/AKT pathway were upstream of mitochondrial ROS production, as febuxostat, and Ly294002 significantly decreased mitochondrial ROS detected by mitoSOX fluorescence in OCP-stimulated BMDM (Fig. 5e). Using JC-10 to assess mitochondrial depolarization, we confirmed that febuxostat prevented OCP-induced membrane changes (Fig. 5f). Specificity of mitochondrial ROS detection by mitoSOX was verified using apocynin (Supplementary Fig. 2).

**Figure 6:**
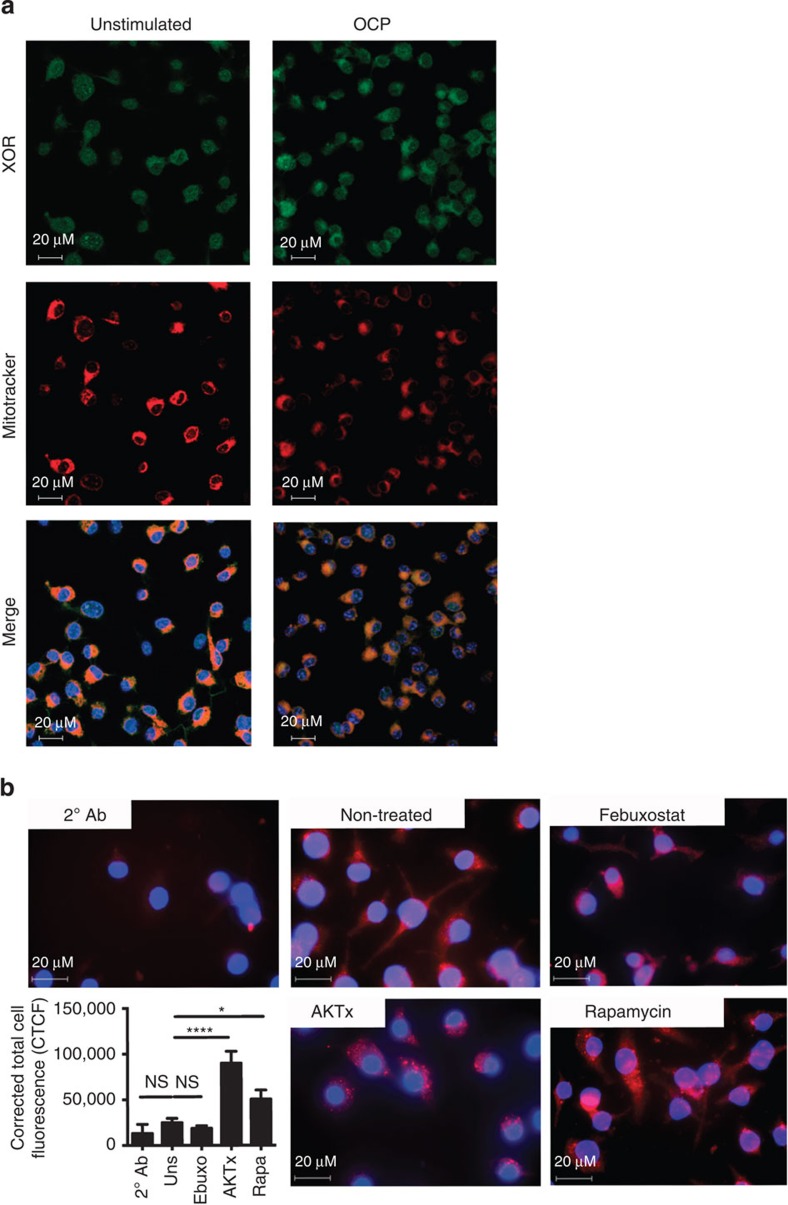
Crystal-mediated XOR activation induces the PI3K–AKT–mTOR pathway and mitochondrial ROS production. (**a**) Colocalization in BMDM of fluorescein isothiocyanate-labelled anti-XOR antibody, Mitotracker and counterstained with 4,6-diamidino-2-phenylindole (DAPI). Images visualized at × 630. Scale bars are indicated. Results are representative of several fields of view with similar results after 6 h of stimulation. (**b**) Autophagic vesicle formation in unstimulated BMDM after 2 h treatment were detected by LC3B Antibody labelled with TRITC, and counterstained with DAPI. Images were visualized at × 400 and are representative of several fields of view with similar results. (**c**) Corrected total cell fluorescence (CTCF) was calculated for each cell in multiple fields of view for LC2B/TRITC red fluorescence. Results are expressed as mean±s.e.m. and significance was determined at *P≤0.05 and ****P≤0.0001 by ANOVA. NP, not performed. NS, not significant.

Interestingly, although XOR co-localized to both the mitochondria and the cytoplasm, OCP exposure did not affect the staining pattern (Fig. 6a). Further investigation saw that XOR was present in both compartments, but with a predominance of the XO form in the mitochondrial fraction. Both the cytoplasmic and the mitochondrial fractions were functional, and febuxostat inhibitable XOR activity was found (Supplementary Fig. 3). Mitophagy and autophagy negatively regulate inflammasome-mediated IL1β secretion^10^,^33^,^34^. Thus, we tested whether XOR inhibition changed the cell’s homeostatic balance to induce autophagic vesicle formation, which would reduce the pool of proIL1β available for cleavage. The degree of autophagy remained unchanged after the treatment of primed, unstimulated BMDM with febuxostat as detected by immunocytochemistry using an antibody specific for the lipidated type-II form of LC3B. Yet, significant increases in autophagy were observed after AKTx and rapamycin administration (Fig. 6b).

These results imply that XO formation is essential for oxidative stress generation, and inflammasome activation via the PI3K–AKT–mTOR pathway, following exposure to crystals.

### Soluble activators of the inflammasome activate XO

**Figure 7:**
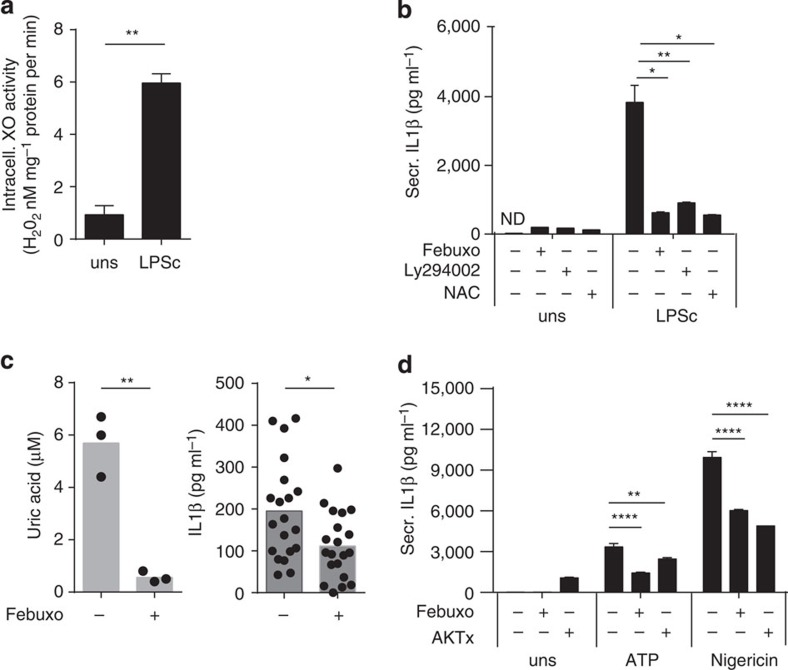
**Inhibition of XOR impairs IL1β secretion triggered by soluble inflammasome inducers and** ***in vivo***. *In vitro* C57BL/6 BMDM (Pam3Cys primed) pretreated with 200 μM febuxostat, *N*-acetyl cysteine (NAC), 10 μM Ly294002 or 10 μM AKTx inhibitor and stimulated with a crude extract of 1 μg ml^−1^ LPS, 2.5 μM nigericin or 5 mM ATP. (**a**) XO activity measured in cell extracts at 1 h. (**b**) IL1β protein secretion measured in BMDM at 6 h. (**c**) Mice injected intraperitoneally with LPSc with or without 1 mM Febuxostat for 6 h. Urate levels and IL1β were measured in cell-free washes (two pooled experiments). (**d**) IL1β protein secretion measured in BMDM after 15 min stimulation for ATP and 6 h for nigericin. Data are representative of at least two experiments. Results are expressed as mean±s.e.m. Statistical significance was determined at *P≤0.05, **P≤0.01 and ****P≤0.0001 by analysis of variance (**b**,**d**) or the *t*-test (**a**,**c**).

As the inflammasome can be activated within macrophages following exposure to soluble PAMPs and DAMPs, we evaluated whether XOR was involved. Primed BMDM, stimulated with LPSc (crude extract of LPS), induced significantly more XO activity as compared with unstimulated controls, and when febuxostat, NAC or Ly294002 was added prior to stimulation there was a significant reduction in secreted IL1β (Fig. 7a,b). As with our *in vivo* crystal-induced peritonitis model, we performed *in vivo* studies in mice to confirm our *in vitro* work. Febuxostat administration, prior to LPSc injection, reduced the urate and IL1β detected in peritoneal washes (Fig. 7c). Finally, IL1β secretion induced by LPS, ATP and nigericin was markedly impaired after febuxostat or AKTx treatment (Fig. 7d).

These results confirm our hypothesis that XOR activation is essential for inflammasome activation and IL1β cytokine secretion.

## Discussion

The formation of ROS by phagocytes is critical for bacterial killing, but it can also regulate cellular signalling cascades during immune activation^35^. The inhibition of NLRP3-inflammasome-mediated IL1β secretion by free-radical scavengers such as apocynin demonstrates the importance of this signalling pathway^36^. Multiple intracellular sources of ROS exist, including NOX complexes, the mitochondria^37^ and last but not least, through activity of the oxidase form of XOR. Although it was initially thought that inflammasome ROS was generated by NOX, this idea was refuted by evidence from studies using human and mice cells deficient for components of the NOX complex.

Our data showed that the XO form of XOR is the major source of ROS in macrophages that mediates NLRP3 inflammasome activation. XOR is an evolutionarily conserved, constitutively expressed enzyme involved in purine degradation that catalyses the conversion of hypoxanthine or xanthine to UA. It is a homodimer; each 145-kDa subunit has three domains: an N-terminal 20-kDa domain containing two Fe–S centres, a central 40-kDa flavin adenine dinucleotide domain and a C-terminal 85-kDa molybdopterin-binding domain. XOR is predominantly a NAD^+^-dependent dehydrogenase (XDH), but upon oxidative stress, it can be converted to the oxygenase form. Both forms generate UA, but the XO form also produces superoxide and hydrogen peroxide. The conversion of XDH to XO can be by irreversible proteolytic cleavage of the 145-kDa protein to an 85-kDa form, or by reversible sulfhydryl modification that changes the electron acceptor sensitivity of the flavin adenine dinucleotide domain through tertiary structure conformational modifications^38^,^39^,^40^.

Our results show that XO inhibition attenuates NLRP3 inflammasome activation, as evidenced by impaired caspase1 and IL1β secretion in macrophages *in vitro* and *in vivo*. There was no effect of febuxostat on activation of the AIM2 inflammasome (Supplementary Fig. 4), indicating that this mechanism may be specific for NLRP3 activation. We used XO inhibitors in our studies rather than siRNA, as we could administer the inhibitors after cellular priming, thus excluding that our results were to due an effect on Mφ priming via TLR stimulation. Although, mutated and deficient XOR mice exist, they die prematurely making *in vivo* experimentation difficult^41^,^42^.

We suggest that the conversion of XOR to XO occurs via reversible oxidation of cysteine residues, rather than proteolytic cleavage of the protein into the 85-kDa form, as we found no change in the molecular weight of XOR when the inflammasome was activated by OCP crystals, and because dithiothreitol treatment of BMDM converted a large proportion of XO activity to XDH. XOR protein was localized to the cytoplasm as well as to the mitochondria by confocal microscopy, so both compartments may participate in ROS generation. As there are no XO-specific antibodies available, we cannot definitively confirm that it is the XO form that is found in both locations. However, cell fractionation experiments showed that both cytoplasmic and mitochondrial fractions had measurable XOR activities, in support of our hypothesis.

We determined that XOR-dependent ROS also activates the AKT–PI3K–mTOR pathway in macrophages^30^,^31^,^32^. Cellular oxidative stress is linked to the AKT–PI3K–mTOR pathway. ROS inactivates the regulatory subunit PTEN (phosphatase and tensin homologue deleted on chromosome 10), which enables downstream activation of AKT, and the mTOR complex 1 refs 43, 44. In our *in vitro* stimulation experiments, febuxostat inhibited the phosphorylation of AKT, reduced mitochondrial ROS and impacted processing and IL1β secretion by caspase1. As autophagy reduces inflammasome-dependent IL1β secretion and is negatively regulated by the AKT pathway^10^,^45^,^46^,^47^, we wondered whether our results were because febuxostat increases autophagy. Yet, we found no evidence of increased autophagy when cells were exposed to febuxostat and concluded that the observed responses were due to impaired ROS generation. Furthermore, febuxostat did not interfere with calcium flux (Supplementary Fig. 5).

We also investigated whether it was UA, the other product of XOR activity, that mediated cellular activation. UA crystals are potent inflammasome activators, yet little is known about the effects of soluble urate on immune responses. Despite observing high intracellular urate levels after crystal stimulation, IL1β secretion remained high in cells from uricase transgenic macrophages, or when extracellular uricase was added to the culture system. Also, macrophages treated with soluble urate failed to show augmented IL1β secretion, even at concentrations seen in clinical hyperuricemia in man. When we combine these results with the observation that there is no clear urate transport mechanism in macrophage, this suggests to us that soluble UA is not responsible for inflammasome activation. However, it is possible that when large amounts of UA are released from dying cells, urate crystals could be formed, which then activate the inflammasome as a particulate stimulus via XOR activation. We have attempted to confirm this by polarizing light microscopy, but were unable to detect urate crystals within dying cells or in the supernatant.

Besides gout, hyperuricemia is also a risk factor for hypertension and cardiovascular disease^48^, but the mechanisms are not well understood. High urate levels can be pro-inflammatory to the endothelium and renal tubules^16^,^48^,^49^. Our current results could explain these associations. We propose that hyperuricemia is an indicator of increased XO activity that leads to enhanced ROS formation and pro-inflammatory cell responses. This will not only affect IL1β secretion from macrophages, but through its actions on the PI3K–AKT pathway, affect other cellular responses. These interactions could account for the associations previously observed in epidemiological studies, and also why XO inhibition by allopurinol has a beneficial effect on hypertension and stroke^50^,^51^,^52^.

On the basis of our results, we suggest that XOR inhibitors may improve conditions that are linked to inflammasome activation, such as atherosclerosis^28^,^52^,^53^. However, careful exploration of their mechanism of action is needed, as UA can be an important antioxidant in the circulation. Current XOR inhibitors inhibit both the XDH and XO forms of XOR, and are used principally to lower urate levels. To overcome this problem, the development of an XO-specific inhibitor that would impact ROS but leave XDH activity untouched is warranted. This would maintain urate levels but decrease the oxidative stress-dependent pathology of chronic inflammatory diseases.

## Methods

### Mice

C57BL/6 mice were purchased from Charles River. Intracellular uricase transgenic-intUOX-Tg mice and littermate controls (on a C57BL/6 background) were from K. Rock (University of Massachusetts, USA)^18^. All experiments and the housing of the mice adhered to the guidelines set by the ‘Service de la consommation et des affaires vétérinaires du Canton de Vaud’ for the humane use of laboratory animals with authorization from the state veterinarian. Female mice used in experiments were 8–10 weeks of age.

### BMDM preparation and maintenance of THP1 cells

Bone marrow cells were incubated for 7 days on Petri dishes with 30% L929 conditioned media, 20% fetal bovine serum (PAA) in Dulbecco’s modified Eagle’s media (Life Technologies) to differentiate into BMDM. BMDM and THP1 cells were stimulated with complete RPMI (cRPMI) media (Life Technologies) with 10% fetal bovine serum, 1% Hepes (Life Technologies) and 1% penicillin–streptomycin (Sigma-Aldrich), or incomplete RPMI with 1% penicillin–streptomycin only).

### Human monocytes and macrophage generation from healthy donors

Peripheral blood was obtained from healthy donors after informed consent. Peripheral blood mononuclear cells were isolated by Ficoll-Hypaque (Life Technologies). Monocytes were purified by magnetic-associated cell sorting by negative selection. Differentiation used 50 ng ml^−1^ of recombinant human macrophage colony-stimulating factor (R&D systems) in cRPMI.

### XO inhibition and stimulation of cells

Sterile, pyrogen-free (≤0.01 EUper 10 mg—by Limulus amebocyte cell lysate assay) OCP, and MSU crystals were synthesized as described previously^54^,^55^. Alum and Hz (Invivogen) were resuspended in PBS and sonicated prior to use. Primary cells (BMDM and huMϕ) were primed with 100 ng ml^−1^ Pam3Cys or 20 ng ml^−1^ ultrapure LPS–LPSup (Invivogen) overnight or left unstimulated in cRPMI. THP1 cells were primed for 3 h with 300 ng ml^−1^ phorbol myristate acetate (PMA). Inhibitors used were: 250 μg ml^−1^ allopurinol (Sigma-Aldrich), 200 μM febuxostat (Teijin Pharma Ltd.), 2.5 mM apocynin (Sigma-Aldrich), 12.5 mM NAC (Sigma-Aldrich), 20 μg ml^−1^ rasburicase (Sanofi), 10 μM Wortmannin (Sigma-Aldrich), 10 μM Ly294002 (Sigma-Aldrich), 10 μM AKTx inhibitor (Calbiochem) and 50 nM rapamycin (Sigma-Aldrich). To ensure that our results were due to the actions of the inhibitor on the cell, our non-treated controls always contained equal quantities of the resuspension vehicle of the inhibitors. For febuxostat, wortmannin, Ly294002 and AKTx this was dimethylsulphoxide (DMSO). Cells were stimulated using 250 μg ml^−1^ OCP and MSU, 500 μg ml^−1^ Alum, 200 μg ml^−1^ Hz and 1 μg ml^−1^ crude LPS (*Escherichia coli* O111:B4—Sigma-Aldrich) for 6 h, unless stated otherwise. Supernatants were collected, the cells harvested and protein or RNA analysed.

### XOR activity and UA determination

Cell lysates were tested for XO activity (AAT Bioquest) and UA quantification (Invitrogen) using fluorescence assays. The amount of fluorescent resorufin is directly proportional to the amount of H_2_0_2_ generated during the respective enzymatic reactions. Fluorescence was measured at Ex_540_ Em_590_ cutoff 590. The manufacturer’s protocols were followed. The results from the *in vivo* OCP peritonitis model were expressed in RFUs and the intracellular XO levels in BMDM were expressed as nM H_2_0_2_ per mg protein per min. The pterin assay to discriminate between XO and XDH activity was performed as previously described^29^. Briefly, cell lysates of BMDM were placed into two different reaction mixes—one that measures XO activity using the conversion of 50 μM pterin to isoxanthopterin, and the other that measures total XOR activity by using methylene blue to replace NAD+ as the electron receptor. Fluorescence was measured at 345/390 nM. The results were calculated as the percentage of XO or XDH activity against the total XOR activity.

### Cytokine and XOR protein quantification

Supernatants or protein lysates from cells were assayed using the IL1β enzyme-linked immunosorbent assay (ELISA) kit (eBioscience). XOR protein quantified using the anti-mouse XDH ELISA kit (USCN life sciences). All manufacturers’ protocols were followed. The results were read at 450 nm using the Spectrax M5e (Molecular Devices). Caspase1 activity was measured using a fluorometric activity test that measures the amount of free 7-amino-4-trifluoromethylcoumarin (AFC) that has been cleaved from YVAD-AFC by active caspase1 within the cell. Fluorescence was detected at Ex 400 nm Em 505 nm (Biovision Inc.). Human caspase1 was measured by ELISA (eBioscience).

### *In vivo* experiments

Female C57BL/6 mice were treated with Allopurinol 500 μg per mouse, Febuxostat 1 mM or rasburicase 20 μg ml−1 or a DMSO vehicle control prior to injection with OCP crystals or LPSc in the peritoneal cavity. PBS alone was administered as a control. After 6 h, mice were sacrificed, the peritoneal cavity washed with PBS and then analysed for urate, extracellular XO activity and IL1β by ELISA (eBioscience).

### SiRNA knockdown of XOR

BMDM were transfected using the Amaxa nucleofector (Lonza AG). BMDM were transfected using either XOR-specific siRNA 5′-CCGAGGATTTCAAACCTTTA-3′ or control siRNA (proprietary sequence-SI03650318) at 300 nM (Qiagen). Knockdown efficiency was determined by quantitative real-time PCR and the XO activity assay.

### Western blot

Cells were lysed in 0.5% NP-40 lysis buffer of 150 mM NaCl, Tris-HCl with EDTA-free protease inhibitor cocktail (Roche), and resuspended with Lammeli buffer. Blots were prepared using polyacrylamide gels, and nitrocellulose membranes using the Bio-Rad II system. Antibodies used were anti-human IL1β (1:1,000 dilution, Cell Signaling Technology), anti-human procaspase-1 (1:500 dilution, gift from R. Solari), Phospho-Akt^Ser473^ (1:2,000, Cell Signaling Technology) or α-tubulin (1:2,000 dilution, Sigma-Aldrich), anti-human XDH antibody (1:2,000 dilution, xAbcam) and anti-rabbit-horseradish peroxidase (HRP), and anti-mouse HRP (1:2.000 dilution, Jackson laboratories). Blots were revealed using the Las2000 system with the Westbright Iris ECL reagent (Advansta). Subcellular fractions were blotted with Anti-XOR (H-110, Santa Cruz Biotechnology, at 1:250 dilution), anti-Tom20 (FL-145, Santa Cruz Biotechnology, at 1:500 dilution) and anti-α-tubulin (Sigma, at 1:5,000 dilution). Images were cropped for figure presentation. Complete images are available as Supplementary Figs.

### ROS assays

Luminol—100 nM and HRP—5 UI ml^−1^ (Sigma-Aldrich) was added concurrently to the stimuli and luminescence measured after 60 min using the Tecan M200. Results were expressed as relative luminescence units. For DHE and MitoSOX experiments, reagents were resuspended in DMSO and cells were loaded with DHE or mitoSOX (Invitrogen) at 5 μM. Fluorescence was determined at Ex_540_ Em_590_. Results expressed as RFUs at end point minus *T*=0. Experiments used opaque tissue culture-treated 96-well plates (Greiner Bio-One), and performed in HBSS (Life Technologies).

### Quantification of AKT phosphorylation

AKT phosphorylation was measured in cell lysates using the InstantOne ELISA (eBioscience). The manufacturer’s instruction was followed. Results expressed as the ratio of phospho-AKT^Ser473^ versus total AKT.

### Mitochondrial membrane potential assay using JC-10

JC-10 enters the mitochondria, where it is retained in multimeric form while the compartment remains polarized. During membrane depolarization the JC-10 is converted to the monomeric form. Briefly, JC-10 (AAT Bioquest) was added to cells coated onto a black microtiter plate. Cells were then stimulated as above in the presence of JC-10. After 5 min, the fluorescence of the multimeric form (Ex540/590) and monomeric form (Ex_490_Em_525_) were measured. Results were expressed using the ratio of 525/590.

### Calcium flux measurement

BMDM were primed with Pam3Cys O/N, washed and loaded with Fluo-4 (Ca^2+^-sensitive fluorometric probe) for 1 h at 37 °C in presence of febuxostat or EDTA (as a positive control for calcium flux inhibition). Baseline fluorescence was measured at 480 nm/520 nm. After stimulation with MSU crystals, fluorescence intensity was measured at 15 s interval over 15 min.

### Subcellular fractionation and western blot

Cellular fractionation of BMDM was performed as previously described^1^. (Briefly, BMDM were harvested, resuspended in hypotonic buffer (100 mM sucrose, 10 mM HEPES and 1 mM EDTA) and passed through a 30-G needle and syringe, 1 in 12 volumes of hypertonic buffer (1.78 M sucrose, 10 mM HEPES and 1 mM EDTA) was added to homogenate, then centrifuged at 700*g* and the supernatant harvested as cytoplasmic fraction.) The precipitated crude mitochondrial fraction was washed, and resuspended with isolation buffer (250 mM sucrose, 10 mM HEPES and 1 mM EDTA). Protein was quantified using the bradford colorimetric assay (BCA) method (Bio-Rad laboratories). Equal protein quantities of the cytoplasmic and mitochondrial fractions were loaded on to an SDS–polyacrylamide gel electrophoresis gel.

### Microscopy experiments

To study autophagy, BMDM were stimulated on glass microscope slides as above, fixed with 98% methanol for 10 min at −20 °C and permeabilized with 1 × PBS and 0.5% Triton X-100 (Sigma-Aldrich). Cells were incubated overnight with LC3B antibody (diluted 1/400 Cell Signaling) in antibody diluent (BD Biosciences) and then stained with biotinylated anti-rabbit antibody (Vector) followed by streptavidin-TRITC (AbD Serotec). Slides were mounted with ProLong Gold Antifade Reagent with 4',6-diamidino-2-phenylindole (Life technologies). Images were taken using Upright Confocal Microscope Leica SP5 Tandem and visualized at × 1,000 magnification. Quantification of autophagy was performed using ImageJ on all experimental conditions. Briefly, the corrected total cell fluorescence was calculated for each cell present within a field of view, and two independent fields of view were analysed with at least 18 cells measured in total. Corrected total cell fluorescence was calculated by measuring the integrated fluorescence density of the area of the cells—(the area of the cell × the mean fluorescence of the background). The results of all of the individual cells were pooled to give the mean and s.e.m. for each condition. This quantification was performed in ImageJ.

Colocalization experiments were performed with BMDM adhered to glass microscope slides and stimulated as above. Cells were stained with Mitotracker Red (Life Technologies) and fixed with 4% paraformaldehyde for 20 min at RT. Cells were permeabilized with 1x PBS and 0.5% Triton X-100 (Sigma-Aldrich), and incubated overnight with anti-XOR Ab (5 ug ml^−1^, Santa Cruz Biotechnology Inc) in antibody diluent (BD Biosciences) followed by incubation with biotinylated anti-rabbit antibody (Vector) and then streptavidin-FITC (eBiosciences). Slides were mounted with ProLong Gold Antifade Reagent with 4,6-diamidino-2-phenylindole (Life technologies). Images were taken using Upright Confocal Microscope Leica SP5 Tandem and visualized at × 630 magnification.

### UA uptake assay

BMDM were incubated with 167 μM of ^14^C UA in uptake buffer (125 mM sodium gluconate, 4.8 mM potassium gluconate, 1.2 mM potassium phosphate, monobasic, 1.2 mM magnesium sulfate, 1.3 mM calcium gluconate, 5.6 mM glucose, 25 mM Hepes, pH 7.3—all purchased from Sigma-Aldrich) for 20 min. Ultima Gold scintillation fluid (PerkinElmer) was added and incubated 2 h for complete cellular lysis. Plate was read on a top count scintillation counter. Results were expressed as the radioactive counts per minute. Cell viability was controlled by Cell Titer Glo reagent (Promega).

### PCR and quantitative real-time PCR

Briefly, cells were placed into Trizol (Invitrogen), RNA was extracted using the DirectZol RNA extraction kit (Zymoresearch) and reverse transcribed into complementary DNA using Superscript II (Invitrogen). Relative expression levels of RNA transcripts were determined using gene-specific primers, SYBR green and the LightCycler 480 system (Roche). Gene-specific primers were Tata-binding protein (*Tbp*): 5′-CCGTGAATCTTGGCTGTAAAC-3′ and 5′-TCCAGAACTGAAAATCAACGC-3′; *Gapdh*: 5′-CTCATGACCACAGTCCATGC-3′, 3′-CACATTGGGGGTAGGAACAC-5′; Urat1: 5′-ACACAGCCAGTCTCTTGATGGAGTG-3′, 3′-CCGTGATGAGCCAGCGTGCC-5′; *Abcg2*: 5′-ACCAGTGTTTTTCCAGTGTGTCAGC-3′, 3′-TCCACCGTCTTCTTCAGTCCTAACA-5′; *Glut9*: 5′-GGGGCAGAGCCTGCAAGGTG-3′, 3′-ACAGGTGACCCCTGCCACCC-5′; XOR: 5′-TAGAAGAAAGTTGGGGCTGTGCGG-3′ and 5′-AGGAGCAGATGGGGGTCAAGCAG-3′. Relative expression levels of genes of interest were calculated using 2^ΔΔCT^ method with *Tbp*, and *Gapdh* as reference genes.

### Graphical representation and statistical analyses

Figures and statistics were generated by Graphpad prism version 6. *In vitro* experiments were performed in replicate. Results expressed as mean and s.e.m. Statistical significance determined using two-tailed parametric *t*-tests or analysis of variance.

## Author contributions

A.I. and N.B. conceived the main experiments, performed the experiments, analysed the data and wrote the manuscript. A.S. analysed the data and wrote the manuscript. J.N., F.M., T.R., D.L., G.S. and J.N.M. contributed additional experimental data.

## Additional information

**How to cite this article:** Ives, A. *et al*. Xanthine oxidoreductase regulates macrophage IL1β secretion upon NLRP3 inflammasome activation. *Nat. Commun*. 6:6555 doi: 10.1038/ncomms7555 (2015).

## Supplementary Material

Supplementary Information Supplementary Figures 1-9

## Figures and Tables

**Table 1:** XOR inhibition impairs OCP-induced IL1**β** secretion in human macrophages.

Macrophages	IL-1β secretion levels (pg ml^−1^, mean±s.e.m.)			
Subject	Priming	Stimulus	Non-treated	Febuxostat
1	Pam3Cys	OCP	228.98±98.57	46.46±12.93^**^
	LPSup	OCP	281.07±38.06	22.32±24.41^***^
2	Pam3Cys	OCP	1,039.75±35.61	467.83±36.43^****^
	LPsup	OCP	315.43±1.16	47.12±2.11^**^
3	Pam3Cys	OCP	1,221.35±20.51	107.43±11.81^****^
	LPsup	OCP	48.23±8.17	Not induced
4	Pam3Cys	OCP	913.21±8.21	954.74±37.85
	LPsup	OCP	377.27±125.29	20.72±21.78^**^

IL1β, interleukin-1β; M-CSF, macrophage colony-stimulating factor; OCP, octacalcium phosphate; PBMC, peripheral blood mononuclear cell; TLR, toll-like receptor; XOR, xanthine oxidoreductase.

Human PBMC-derived monocytes were differentiated into Mφσ with 50 ng ml^−1^ M-CSF. Cells were primed with TLR agonists, pretreated with febuxostat 200 μM and stimulated with OCP 250 μg ml^−1^ for 6 h. IL1β in supernatants was measured. Results expressed as the mean±s.e.m. of independent stimulations. Statistical significance determined at **P≤0.01, ***P≤0.005, ****P≤0.0001 by the *t*-test.
