# Supplementary material for: Xanthine oxidoreductase regulates macrophage IL1β secretion upon NLRP3 inflammasome activation
Source: Nat Commun. 2015 Mar 24;6:6555. doi: 10.1038/ncomms7555 (PMC4382995; doi:10.1038/ncomms7555)
Supplement: Supplementary Information — Supplementary Figures 1-9 [file ncomms7555-s1.pdf]

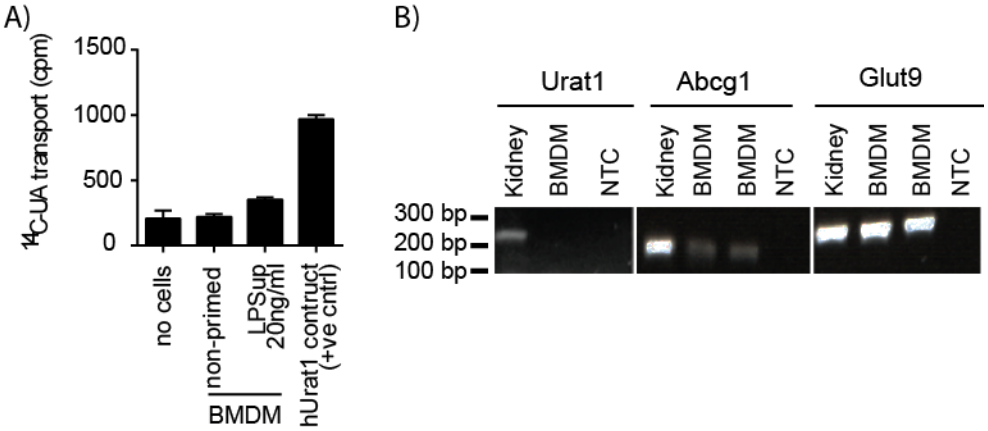

Supplementary figure 1. Macrophages do not take up soluble urate and have minimal urate transport expression. A) Radiolabeled Urate uptake assay of BMDM versus control human Urat1 transfected Hek293 cells, results expressed as counts per minute (cpm). B) RNA transcript expression profile of known urate transporters Urat1, Abcg1, and Glut9 in BMDM versus whole kidney lysate control, using cDNA reverse transcribed from RNA, and elaborated on a 1% agarose gel. Results were expressed as mean ± SEM.

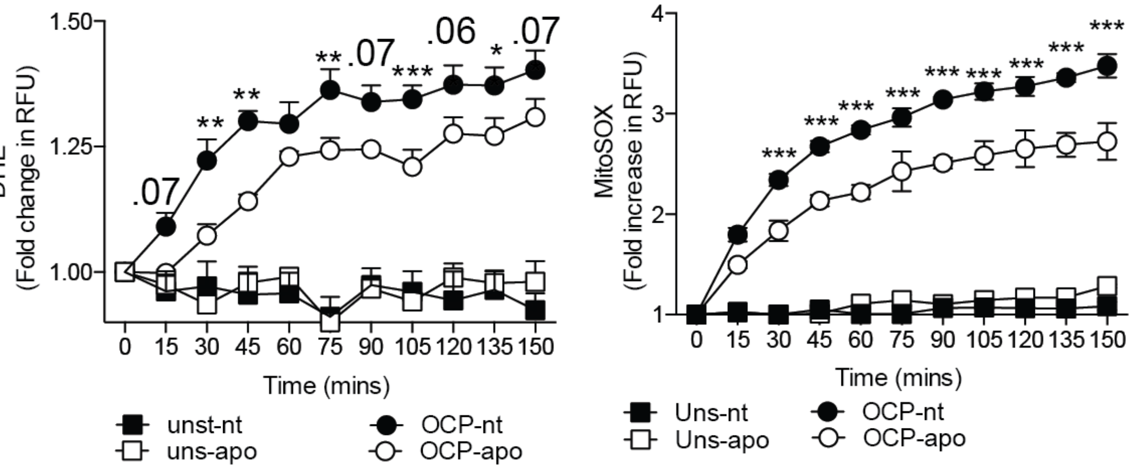

Supplementary figure 2: Apocynin decreases the fluorescence signals following BMDM stimulation in the presence of DHE or MitoSOX. *In vitro* BMDM (P3C primed) were pretreated with apocynin 5mM, and stimulated with OCP. A) Superoxide measured by Dihydroethidium (DHE), results expressed as fold increase in relative fluorescence units-RFU at T= 120mins over T=0. B) Mitochondrial ROS detected by MitoSOX, results given as RFU at T=60 minutes over T=0. Results expressed as mean ± SEM. Significance determined at *p≤0.05, **p≤0.01, ***p≤0.005, ****p≤0.0001, by ANOVA.

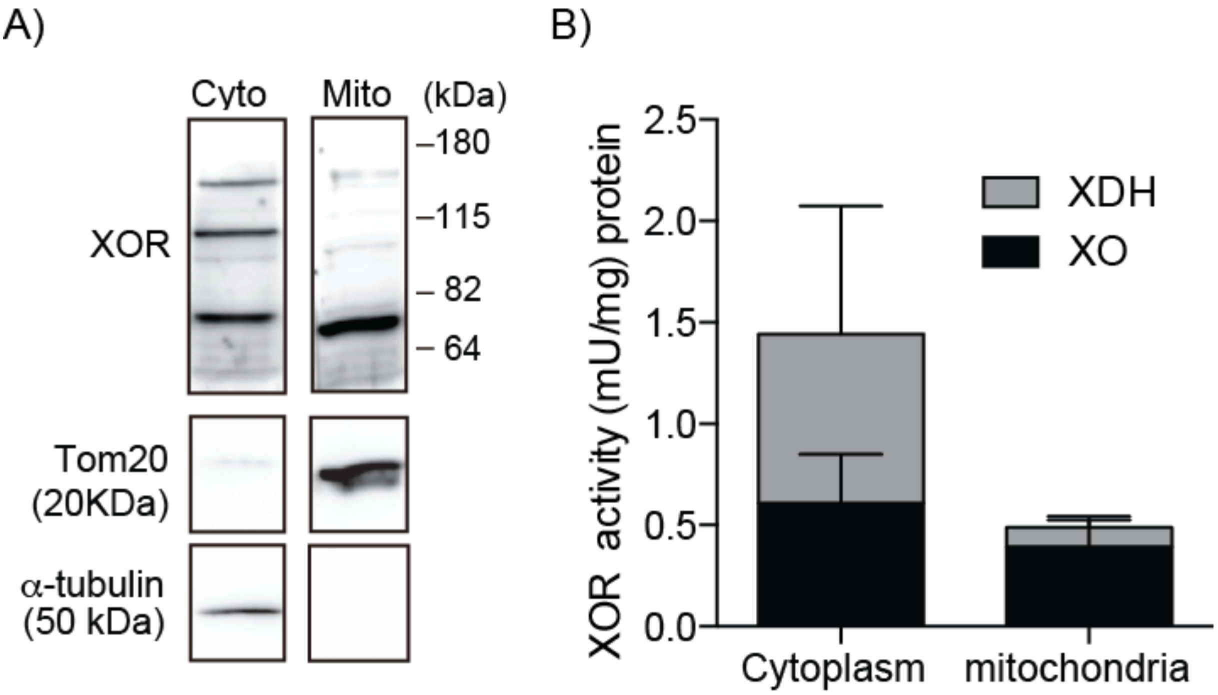

Supplementary figure 3: Functional XOR is present in both the mitochondrial and cytoplasm of macrophages. Unstimulated, primed BMDM were harvested and subcellular mitochondrial and cytoplasmic fractions were isolated. A) Fractions were analyzed with anti-XOR Ab, anti-Tom20 and anti-α–tubulin by western blot. B) XO vs XDH activity was determined using the pterin assay. Data represents at least two independent experiments with the mean ± SEM.

A. BMDM

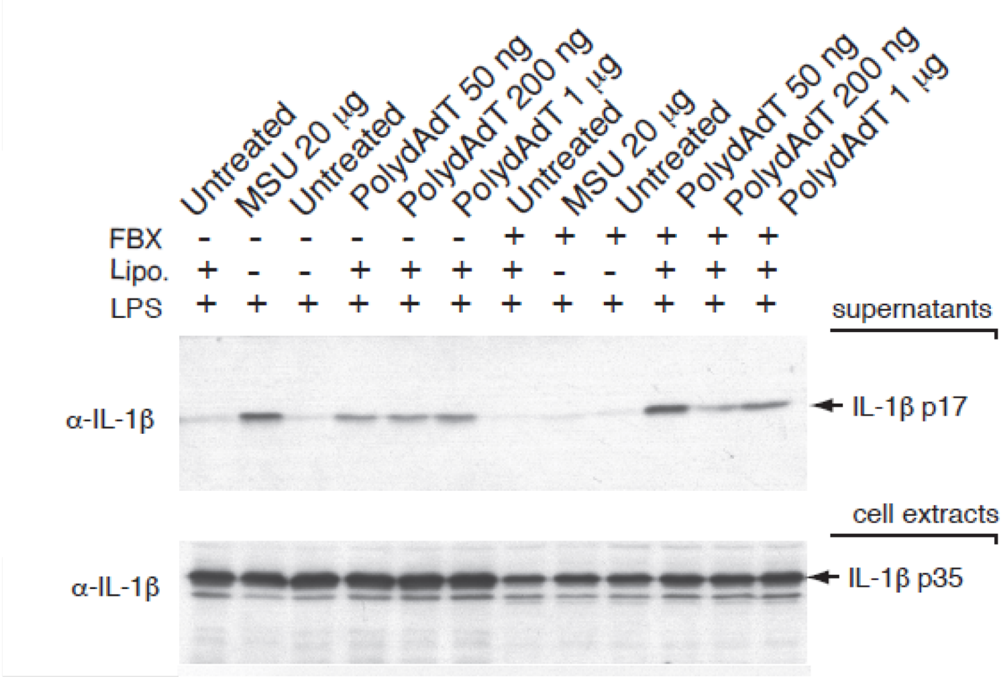

B. THP-1

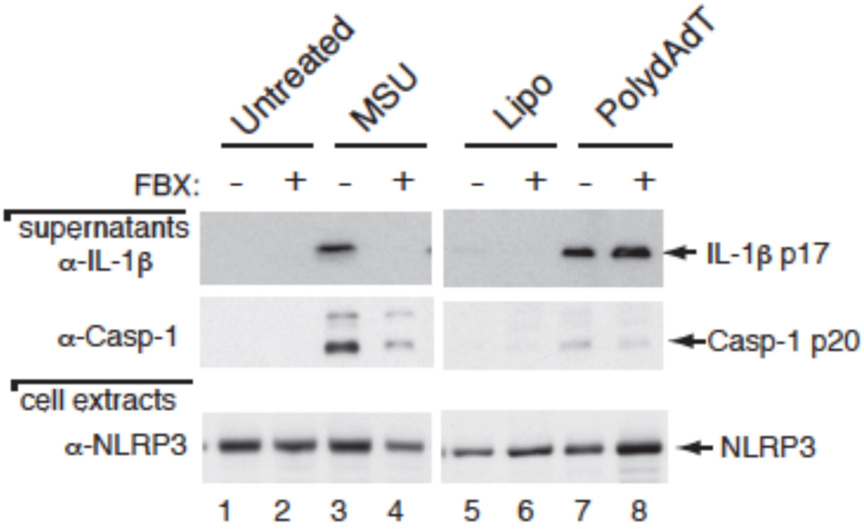

Supplementary figure 4: Febuxostat does not inhibit AIM2 inflammasome activation.
A. BMDMs were stimulated by polydAdT (at three different concentrations) in the blot of IL1β from supernatants and cell extracts showed no inhibition of IL1β secretion when AIM2 was activated. B. THP-1 cells were stimulated with MSU and polydAdT in the presence and absence of febuxostat. Caspase-1 and IL1β released into the supernatant was revealed by western blot. AIM2 stimulation was not affected by febuxostat. Images have been cropped for presentation. Full size images are presented in supplementary Fig 6 and 7.

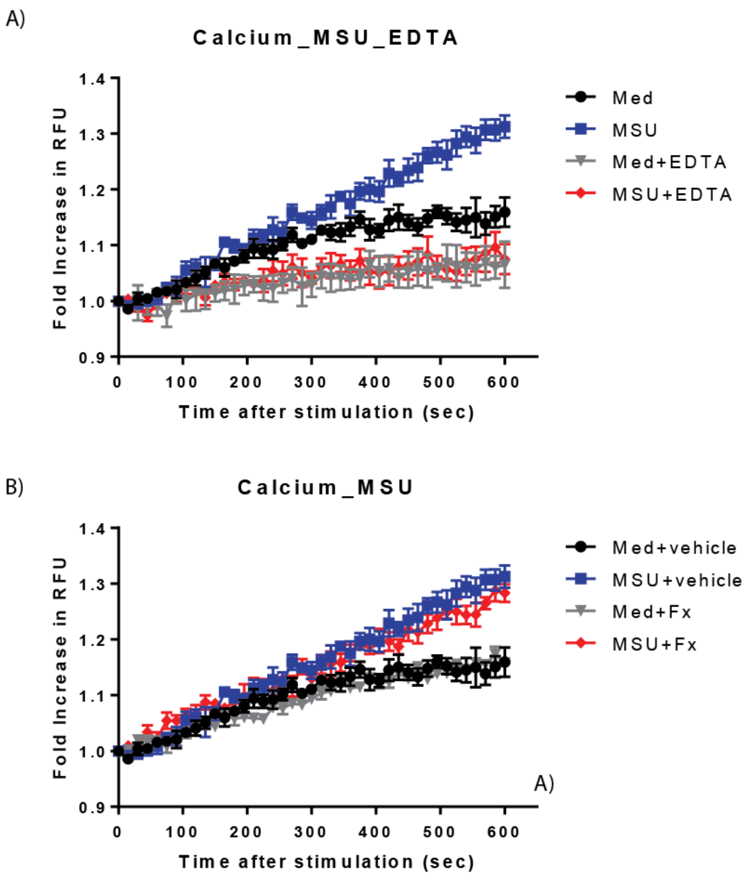

Supplementary figure 5: Calcium flux on MSU stimulation is not inhibited by febuxostat. Ca2+ is mobilized during MSU stimulation. Febuxostat had no effect on Ca2+ flux, whereas as expected EDTA completely blocked MSU-induced Ca2+ mobilization. Data is expressed a.s mean ± SEM.

Uncroped images for Supplementary Figure 4a

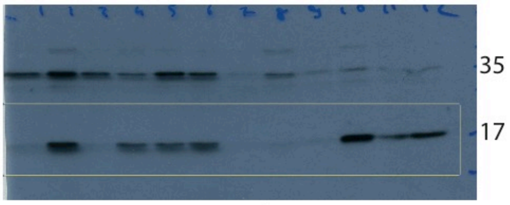

BMDM SN , WB anti-IL-1β

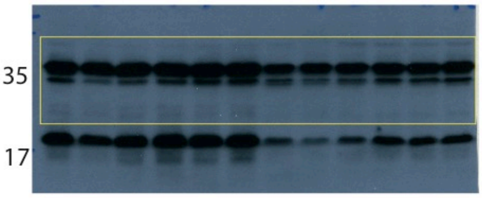

BMDM XT , WB anti-IL-1β

Uncroped images for Supplementary Figure 4b

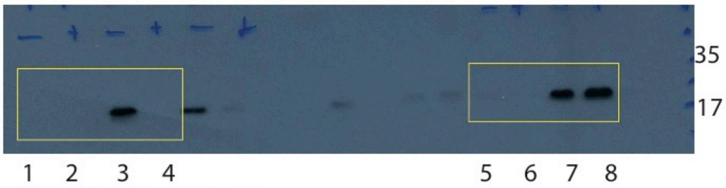

THP1 SN , WB anti-IL-1β

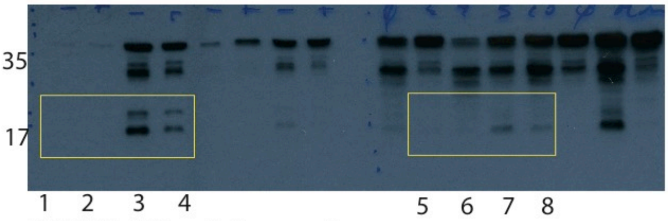

THP1 SN , WB anti-Caspase-1

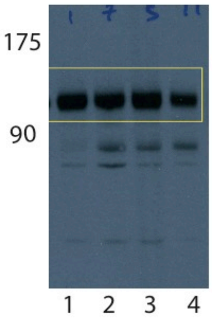

THP1 XT , WB anti-NLRP3

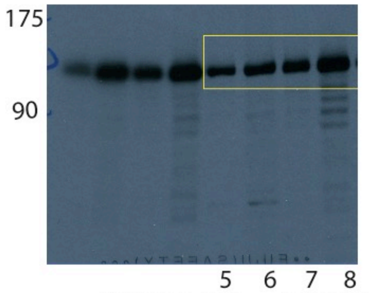

THP1 XT , WB anti-NLRP3

Supplementary Figure 6: Full sized uncropped images of western blot of THP-1 cells in Supplementary Figure 4A and B.

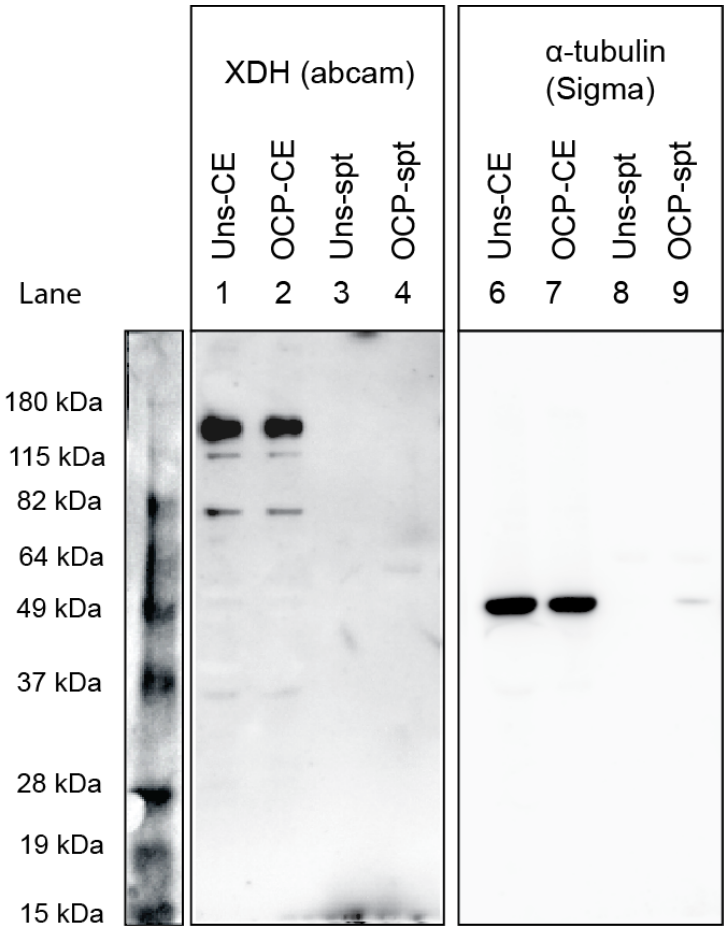

Full sized image of western blot for figure 4 D

Supplementary Figure 7: Immunoblot XDH and tubulin

A) anti- human IL1β (cell signalling 2021s)

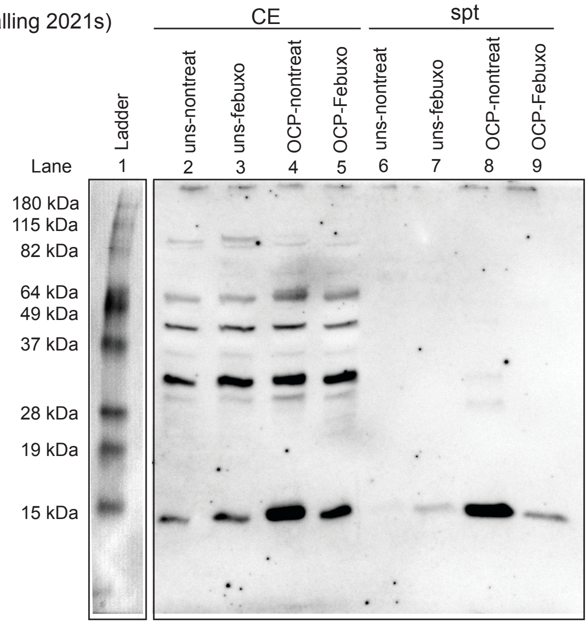

B) anti-human Procaspase1 (gift from R.Solari)

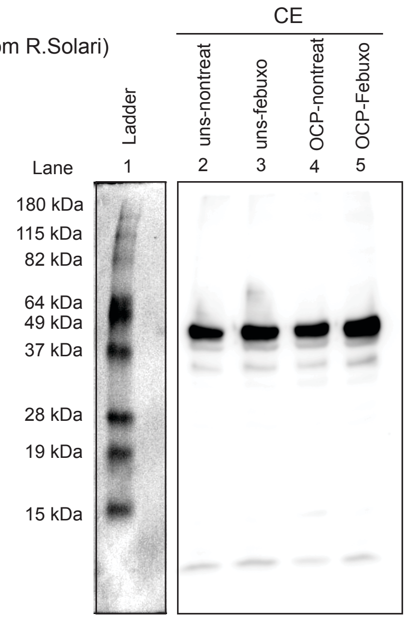

Supplementary Figure 8: Immunoblot for Figure 1E

Figure S9A XOR immunoblot

XOR immunoblot

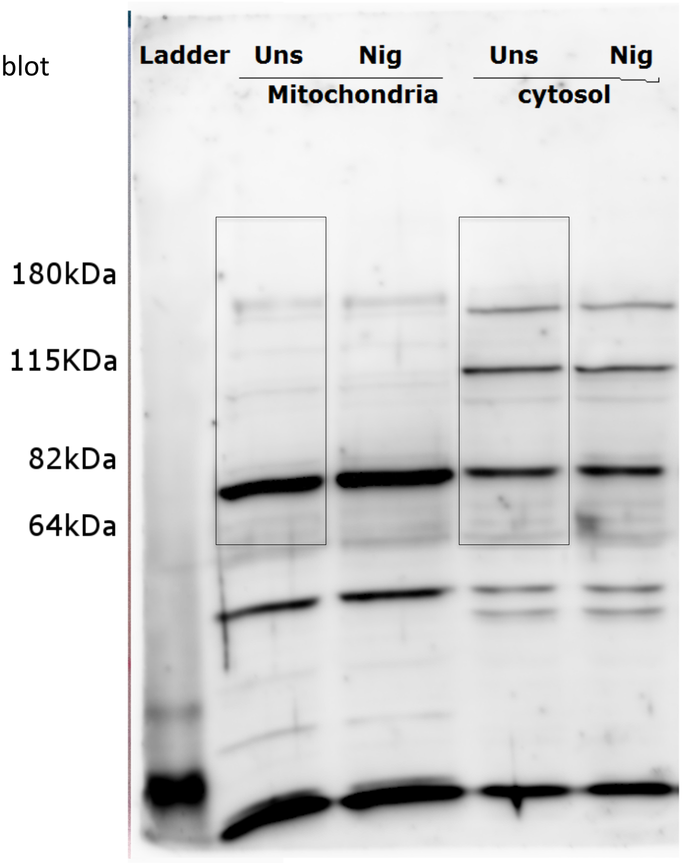

Supplementary Figure 9A: Uncropped immunoblots for preparation of Supplementary Figure 2

Tom20 immunoblot

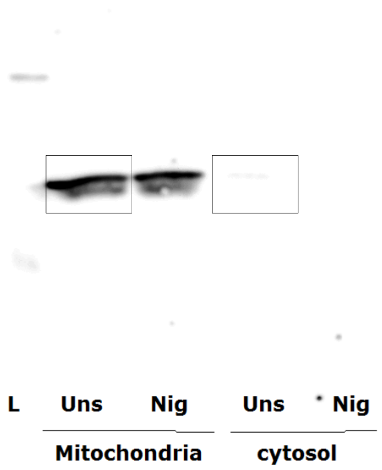

Supplementary Figure 9B: Tom20 immunoblot

Tubulin immunoblot

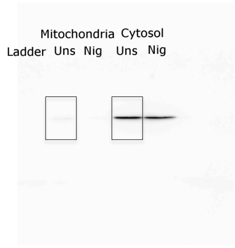

Supplementary Figure 9C: Tubulin immunoblot
